# Single-molecule magnetic tweezers to unravel protein folding dynamics under force

**DOI:** 10.1007/s12551-025-01274-1

**Published:** 2025-02-08

**Authors:** Rafael Tapia-Rojo

**Affiliations:** Department of Physics and Centre for the Physical Science of Life, King’s College London, London, UK

**Keywords:** Single-molecule force spectroscopy, Magnetic tweezers, Protein folding, Protein nanomechanics

## Abstract

Single-molecule magnetic tweezers have recently emerged as a powerful technique for measuring the equilibrium dynamics of individual proteins under force. In magnetic tweezers, a single protein is tethered between a glass coverslip and a superparamagnetic bead, and by applying and controlling a magnetic field, the protein is mechanically stretched while force-induced conformational changes are measured by tracking the vertical position of the bead. The soft trap created by the magnetic field provides intrinsic force-clamp conditions, which makes magnetic tweezers particularly well-suited to measure protein conformational dynamics. Traditionally employed to study DNA due to their initially low spatial and temporal resolutions, magnetic tweezers instrumentation has experienced significant progress in recent years. The development of high-speed cameras, stronger illumination sources, advanced image analysis algorithms, and dedicated chemical functionalization strategies, now allow for high-resolution and ultra-stable experiments. Together with their ability to apply and control low forces, magnetic tweezers can capture long-term equilibrium protein folding dynamics, not possible with any other technique. These capabilities have proven particularly valuable in the study of force-sensing protein systems, which often exhibit low mechanical stabilities that are challenging to measure with other techniques. In this review, we will discuss the current status of magnetic tweezers instrumentation for studying protein folding dynamics, focusing on both the instrumental aspects and methodologies to interpret nanomechanical experiments.

## Introduction

Proteins are central to cellular processes, virtually in charge of most molecular mechanisms underpinning life. Traditionally, protein function has been univocally linked to their ability to fold into specific three-dimensional structures—the folded native state. Despite significant advances in our understanding of protein folding since Levinthal’s paradox was first posed (Levinthal 1968; Zwanzig et al. 1992), how proteins can spontaneously fold into their unique native state within biological times remains an open question in the field of biophysics. More recently, this classic, static view of protein structure has been challenged, shifting towards an understanding that proteins are intrinsically dynamic molecules: in addition to the importance of the folded three-dimensional architecture of the native state, proteins undergo conformational changes—which can occur stochastically or be externally triggered—and such conformational plasticity can indeed be critical to their function (Bowman 2024; Nam and Wolf-Watz 2023).

The advent of single-molecule force spectroscopy techniques in the 1990s revolutionized the study of protein dynamics (Mora et al. 2020; Neuman and Nagy 2008). Here, mechanical forces are employed to perturb the folded state and trigger conformational changes that can be directly captured at the individual protein level, gaining access to molecular events that were previously obscured by the ensemble averaging inherent to bulk biochemical methods. In addition to the use of force spectroscopy techniques as a protein biophysical characterization method, mechanical forces are now recognized as critical regulators of protein function in multiple biological processes (Yusko and Asbury 2014). Muscle proteins, such as titin, have long been prototypical examples in this regard, which motivated the use of titin as a classic model system in many initial force spectroscopy studies (Kellermayer et al. 1997; Rief et al. 1997). More broadly, the emerging field of mechanobiology has lately highlighted the critical role of mechanical forces in regulating multiple biological processes, underscoring the importance of understanding how proteins respond dynamically to mechanical force (Alegre-Cebollada 2021; Beedle and Garcia-Manyes 2023).

In single-molecule force spectroscopy techniques, an individual protein is tethered between a substrate and a force probe, so that, upon mechanical stretching—using forces in the piconewton (pN) range—the protein’s conformational dynamics in terms of end-to-end extension changes (here in the nm range) can be measured as a function of force. There are three main force spectroscopy techniques: atomic force microscopy (AFM), optical tweezers, and magnetic tweezers, each based on different working principles and with different strengths and limitations. AFM is particularly well-suited for applying high forces, enabling rapid characterization of the mechanical stability of proteins, particularly those unfolding at forces above ~20 pN (Hoffmann and Dougan 2012; Popa et al. 2013b). Optical tweezers, on the other hand, allow manipulation of low forces, of just a few pN, making them useful for probing protein folding transitions, which generally occur at these lower forces (Bustamante et al. 2021). Both AFM and optical tweezers, however, face stability limitations, as the intrinsic mechanical drift restricts the measuring timescales to a few seconds or minutes at best (Popa et al. 2013a).

Magnetic tweezers were traditionally considered a lower resolution technique, used primarily for studying nucleic acids like DNA, which are stiffer molecules responding elastically to force through larger, easier-to-capture conformational changes (Smith et al. 1992; Strick et al. 1996). However, this technology has seen significant improvements in recent years, which have now positioned magnetic tweezers as a powerful technique for studying protein mechanics at the single-molecule level (Choi et al. 2019; Löf et al. 2019; Popa et al. 2016; Tapia-Rojo et al. 2019; Zhao et al. 2017). This responds to a combination of advances, including the development of high-speed image tracking devices, and the improvement in stable and specific chemical anchoring strategies. In contrast to AFM and optical tweezers, magnetic tweezers offer intrinsic passive force-clamp conditions, a key advantage that enables direct manipulation of the pulling force while the protein conformational dynamics are measured. Moreover, magnetic tweezers offer the possibility of doing multiplexed measurements that increase the experimental yield (Löf et al. 2019; Popa et al. 2016) and are a naturally stable technique, allowing long-term measurements of protein dynamics, hence overcoming the main limitation of the other two force spectroscopy techniques. Altogether, magnetic tweezers have become now a particularly suitable technique to probe protein dynamics under force, opening up new avenues of research not accessible with AFM or optical tweezers.

In this review, we will discuss modern applications of single-molecule magnetic tweezers instrumentation to the study of protein folding dynamics. The manuscript is organized into three main sections. The “Magnetic tweezers instrumentation” section will cover the fundamentals of magnetic tweezers instrumentation, including the image analysis algorithms, force application and calibration methods, and chemical anchoring strategies. While the specifics of magnetic tweezers instrumentation can vary greatly amongst different lab implementations, this section aims to provide an overview of the core principles and most common experimental approaches. The “Understanding protein folding dynamics with magnetic tweezers” section will delve into the physics of protein folding under force, providing the theoretical foundation for designing and interpreting magnetic tweezers experiments. We will introduce a simple free energy landscape model that captures the basic properties of protein folding dynamics under force, before reviewing the most established analytical frameworks for analyzing protein (un)folding dynamics. Finally, the “Applications of magnetic tweezers to study protein dynamics under force” section will discuss recent studies that highlight the use of magnetic tweezers in exploring protein folding dynamics, with a focus on proteins that function in a mechanical environment.

## Magnetic tweezers instrumentation

### Magnetic tweezers in a nutshell

**Fig. 1:**
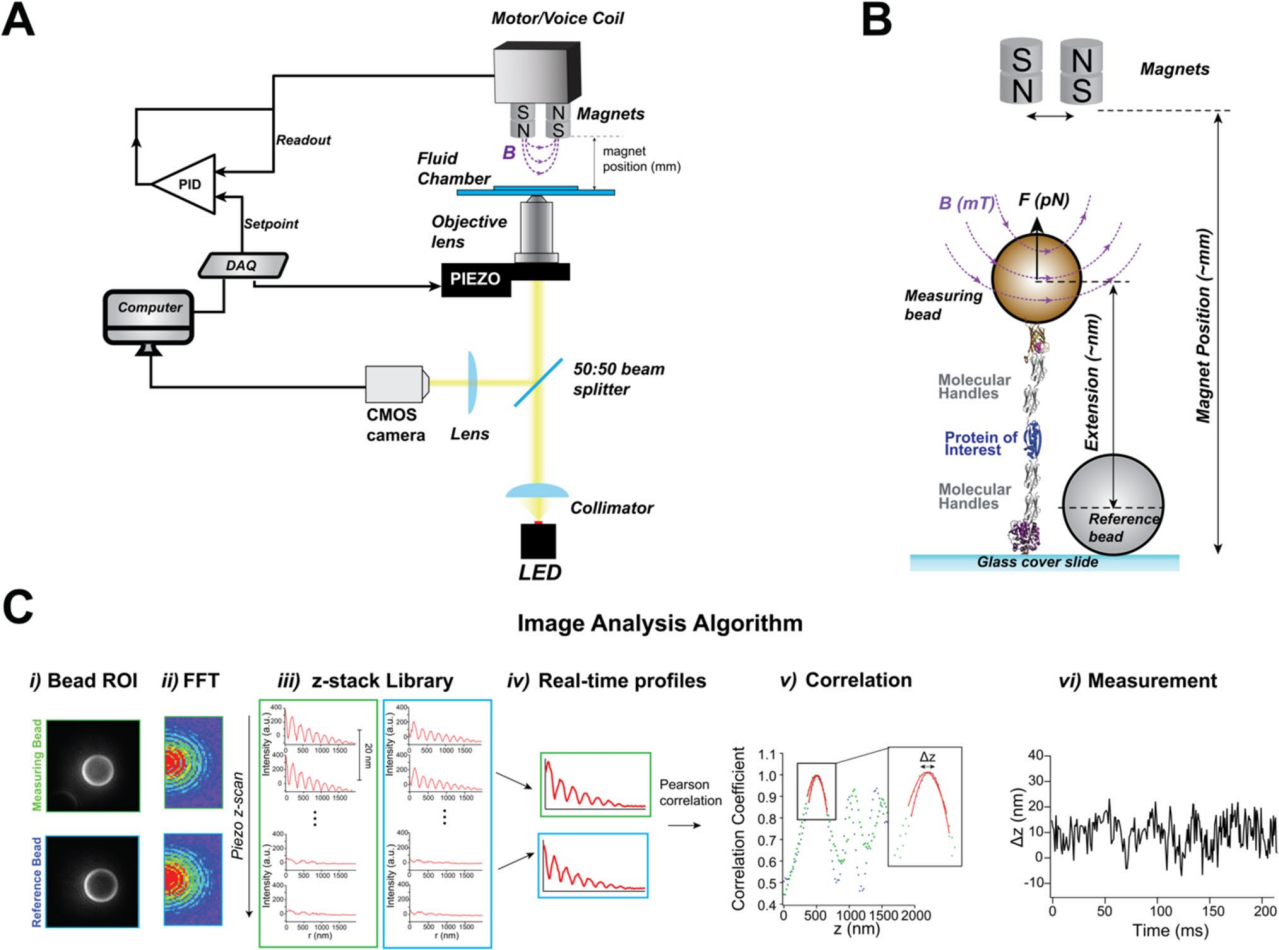
Schematics of a magnetic tweezers setup and experiment. **A** Schematics of a typical magnetic tweezers setup, involving an inverted microscope, a piezo-mounted objective lens, and a pair of permanent magnets mounted on a DC motor or voice coil, placed above the fluid chamber. Keeping the magnet position under electronic feedback using a PID circuit ensures accurate position and movement of the magnets. **B** Schematics of a magnetic tweezers experiment for measuring protein dynamics. A protein construct is anchored between a glass cover slide and the superparamagnetic (measuring) bead. By comparing the relative position of the magnetic and a reference bead attached to the glass, the protein extension is monitored. The magnets are positioned at distances of ~ mm, much larger than the molecular extension, creating a very soft trap that provides passive force clamp conditions. **C** Workflow of the image analysis algorithm. The beads region of interest (ROI) are imaged with the brightfield microscope (*i*), and the Fourier transform of the ROIs is calculated (*ii*). A piezo scan is done at the beginning of the experimen (iii), where the radial profiles for the measuring and reference beads are calculated by integrating the pixel intensity of the FFTs over constant radial positions. In this case, the piezo takes 100 radial profiles spaced by 20 nm. During the experiment, the real-time radial profiles are calculated (*iv*) and correlated with the z-stack library (*v*), allowing measurement of the beads’ position as the distance between the peaks of the Gaussian fits (*vi*)

Compared to the other two main single-molecule techniques—atomic force microscopy (AFM) and optical tweezers—magnetic tweezers are perhaps the most straightforward regarding both working principles and instrumental design (Neuman and Nagy 2008)—and also the most cost-effective one. This simplicity partly explains why most research laboratories rely on custom instruments and the lack of major commercially available magnetic tweezers instruments. Despite the absence of standardized magnetic tweezers designs and the large variability in configurations across different laboratories, all magnetic tweezers instruments share two fundamental components: a bright field microscope and a mechanism to apply and control a magnetic field (Fig. 1A).

In magnetic tweezers, a single molecule is tethered between a glass coverslip and a micron-sized superparamagnetic bead (Fig. 1B). When a magnetic field is applied, it exerts a pulling force on the bead, thereby stretching the tethered molecule. Simultaneously, the conformational dynamics of the molecule—in terms of end-to-end extension changes—are tracked by monitoring the bead’s movement along the *z*-axis using an image-analysis algorithm. The magnetic field can be generated by using a pair of permanent magnets (Chen et al. 2011; Choi et al. 2019; Dulin et al. 2015; Löf et al. 2019; Popa et al. 2016; Smith et al. 1992), an electromagnet (Fisher et al. 2006; Tapia-Rojo et al. 2019), or more sophisticated magnet/electromagnet arrangements (Gosse and Croquette 2002).

Bead tracking typically involves bright-field microscopy to capture the interference or diffraction rings of the superparamagnetic bead, along with those of a non-magnetic reference bead firmly attached to the glass coverslip. The vertical position of the magnetic bead is compared to that of the reference bead to measure extension changes in the molecule, which also cancels out low-frequency drift, critical to the hallmark stability of magnetic tweezers.

In the following subsections, we will explore the instrumental details of modern magnetic tweezers instrumentation.

### The microscope design and bead-tracking algorithm

Magnetic tweezers rely on precise video-tracking of the bead position, typically using an inverted bright-field microscope paired with a complementary metal-oxide semiconductor (CMOS) camera (Fig. 1A). A non-coherent point LED source, collimated to achieve uniform illumination, is commonly used, providing the bright images necessary for accurate tracking. Unlike AFM or optical tweezers, which use fast photodetectors to capture extension changes, the image-analysis-based approach in magnetic tweezers has historically imposed limitations on the temporal resolution. Nevertheless, recent advances in high-speed CMOS sensors, combined with the use of more powerful illumination sources and sophisticated image analysis and processing algorithms (Huhle et al. 2015; van Loenhout et al. 2012), have significantly pushed the resolution limits of magnetic tweezers into the millisecond or even microsecond range (Dulin et al. 2015; Lansdorp et al. 2013).

The vertical position of the measuring bead relative to that of the reference one is determined by tracking their diffraction or interference rings (depending on whether top or bottom illumination is used, respectively), which undergo radial expansion as the beads move away from the focal plane. The ring patterns are pre-calibrated at the beginning of the experiment using a nanopositioning piezo scanner, which moves the objective lens to build a *z*-stack library of ring patterns, spaced by nm-scale piezo displacements. Using a pixel-addressing algorithm on the Fourier transform of the bead’s image, the radial profiles characteristic for each piezo position are calculated by integrating the pixel intensity across constant radial positions. During an experiment, to convert ring pattern changes into spatial displacements, the real-time radial profiles of both the measuring and reference beads are correlated with the pre-recorded profiles in the *z*-stack library, which allows calculating the magnetic bead’s relative vertical position from Gaussian fits to the Pearson-correlation vectors. The piezo is also employed to adjust the focal plane of the reference bead, correcting for drift and enabling long-term measurements. Figure 1C shows the standard image-analysis pipeline in magnetic tweezers.

The spatial resolution achievable in magnetic tweezers depends on various factors beyond instrumental design. The magnitude of the molecular fluctuations is inversely proportional to the applied force and directly related to the contour length of the molecular construct (Stannard et al. 2021). At very low forces, large molecular fluctuations occur, which effectively reduce the signal-to-noise ratio. Using short protein-based constructs may help improve the signal-to-noise ratio compared to longer DNA-based tethers, which can introduce more background fluctuations—since the magnitude of the molecular fluctuations depends directly on the contour length of the full molecular construct (Stannard et al. 2021). Additionally, the image analysis algorithm can introduce some artefactual noise—for instance, due to inaccuracies in the determination of the radial profiles or the fits to the correlation vectors—which can further limit the spatial resolution. However, unlike molecular noise, this instrumental noise is independent of the applied force or the conformational state of the protein under study, so it can often be subtracted using a deconvolution procedure if the associated point-spread function is estimated (Tapia-Rojo et al. 2024a).

### Applying and controlling the pulling force

In magnetic tweezers, mechanical forces are applied to superparamagnetic beads using calibrated magnetic fields. These beads have a diameter in the micron (µm) range and consist of a polystyrene matrix embedded with iron oxide nanoparticles, which give them their superparamagnetic properties. Various suppliers offer these beads with different surface coatings that facilitate easy conjugation with the biomolecule of interest (see “Design of protein constructs and anchoring chemistry” section).

Under a magnetic field $\overrightarrow{B}$, the force $\overrightarrow{F}$ acting on a magnetic particle, in this case, the superparamagnetic bead, can be expressed as follows:

1 $$\begin{array}{c}\overrightarrow{F}=\left(\overrightarrow{m}\bullet \overrightarrow{\nabla }\right)\overrightarrow{B}\end{array}$$

where $\overrightarrow{m}$ is the magnetic moment of the bead. For a superparamagnetic bead in a weak magnetic field, it can be described as follows (Shevkoplyas et al. 2007):

2 $$\begin{array}{c}\overrightarrow{m}={\overrightarrow{m}}_{0}+V{\chi }_{b}\frac{\overrightarrow{B}}{{\mu }_{0}}\end{array}$$

where *V* is the bead’s volume, ${\chi }_{b}$ is its magnetic susceptibility (which can be determined from a magnetization curve), ${\mu }_{0}$ the permeability of the vacuum, and ${\overrightarrow{m}}_{0}$ the initial magnetization of the bead. For a superparamagnetic bead (or any paramagnetic material), ideally ${\overrightarrow{m}}_{0}=0$; however, in practice, this term cannot be neglected, as it has been previously demonstrated (Pamme 2006; Shevkoplyas et al. 2007). Therefore, the force acting on a superparamagnetic bead can be expressed as follows:

3 $$\begin{array}{c}\overrightarrow{F}=\left({\overrightarrow{m}}_{0}\bullet \overrightarrow{\nabla }\right)\overrightarrow{B}+\frac{V{\chi }_{b}}{{\mu }_{0}}\left(\overrightarrow{B}\bullet \nabla \right)\overrightarrow{B}\end{array}$$

Thus, the magnetic force depends not only on the magnetic field but also on its gradient. To achieve a purely pulling force $\overrightarrow{F}=F\widehat{z}$ (which is critical to avoid lateral pulling components that complicate the interpretation of the pulling experiment), it is necessary to operate in a region where the magnetic field along the *x* and *y* components is constant, ensuring there are no lateral field gradients. This can be accomplished by using a pair of permanent magnets in an antiparallel configuration and working below the gap space between them. Importantly, the maximum applicable force depends on the width of the gap, since separating the magnets leads to less steep gradients (Yu et al. 2014). In permanent magnet configurations, potent rare-earth magnets like N52 are often used, which exhibit very high surface fields (~ 2 T), allowing for forces of up to 120 pN when using the 2.8 µm M270 beads (Popa et al. 2016) or even > 400 pN with the larger M450 beads (Alonso-Caballero et al. 2021a, b).

Electromagnet configurations have also been implemented in magnetic tweezers instrumentation, often employing sharp electromagnet tips (L. Chen et al. 2015a, b; Liu et al. 2009). These tips can generate exceptionally high magnetic fields and steep gradients, significantly extending the range of achievable forces to several hundredths of pN or even nN. However, they are often unpractical in the study of protein nanomechanics due to calibration issues stemming from the steep gradient and also require very high electric currents, which necessitate cumbersome cooling systems. Recently, magnetic tape heads have emerged as an alternative electromagnet configuration in magnetic tweezers instrumentation (Tapia-Rojo et al. 2019). Originally developed for high-density magnetic recording (Buschow et al. 2012), these devices have been refined over time to achieve large magnetic fields that can be modulated across a broad bandwidth—a feature that has now found significant utility in the field of force spectroscopy (Tapia-Rojo et al. 2020a, 2019). While magnetic tape heads may achieve lower magnetic fields than permanent magnets (~0.5 T under saturation conditions), they have a very narrow gap (~25 µm) that creates sharp gradients, enabling the application of forces comparable to those achievable with common permanent magnet configurations.

Compared to other force spectroscopy instruments, magnetic tweezers have the unique advantage of their intrinsic force-clamp conditions. This means they allow for direct manipulation of the pulling force, as the magnetic fields change over a length scale (~µm) much larger than the spatial scale over which the beads move (~nm). Thus, magnetic tweezers offer the natural ensemble for measuring molecular conformational dynamics, providing direct control of the intensive variable (force) while the extensive variable (extension) is measured. While applying a constant force load in magnetic tweezers only requires keeping the magnets at a constant position, changing the force presents practical challenges, necessitating the physical displacement of the magnets. This is often achieved by mounting the magnets on a DC motor, or a voice-coil system, which, when maintained under electronic feedback, enables accurate displacements and, hence, force manipulation. DC motors are typically slower (force changes over a few seconds) and more limited in the kind of displacements they can do, while voice-coils are more versatile and faster (~100–200 ms). The use of magnetic tape heads mitigates these limitations as force is directly controlled by the electric current, allowing for force modulations over a bandwidth of several kHz, provided that the tape head is mounted at a fixed position (Tapia-Rojo et al. 2019).

### Calibration of magnetic tweezers

Force in magnetic tweezers instrumentation is calibrated by establishing a direct relationship between the control parameter (i.e., the distance between the magnets and the superparamagnetic bead or the electric current applied to the electromagnet at a given distance) and the pulling force exerted on the measuring bead. This approach requires prior knowledge of how the magnetic field depends on the control parameter, allowing for the calculation of the magnetic force acting on the bead (Eq. 3).

For arrangements involving permanent magnets, this relationship depends on the magnet’s geometry, which often necessitates numerical approaches that can be challenging to compute accurately. Consequently, phenomenological expressions are frequently assumed, the simplest one of which involves an exponential dependence on the control parameter (Popa et al. 2016):

4 $$\begin{array}{c}F\left(MP\right)=A{e}^{-B\bullet \left(MP\right)}\end{array}$$

where *F* is the force acting on the bead, *MP* is the magnet position (or distance between the magnets and bead), and *A* and *B* fitting parameters, which depend on the magnets’ and bead’s properties (geometry, material, etc.). Double exponential corrections to this phenomenological relationship have been proven to be necessary to account for saturation effects or other nonlinearities that may arise over the high force range(Chen et al. 2011; Yu et al. 2014).

In the case of electromagnet-based setups, particularly those using tape heads, it is possible to derive an analytical expression for the magnetic force as a function of the electric current and distance (Tapia-Rojo et al. 2019). In particular:

5 $$\begin{array}{c}F\left(z,I\right)=A{I}^{2}{\text{tan}}^{-1}\left(\frac{{~}^{g}\!\left/ \!{~}_{2}\right.}{z}\right)\frac{1}{1+{\left(\frac{z}{{~}^{g}\!\left/ \!{~}_{2}\right.}\right)}^{2}}+BI\frac{1}{1+{\left(\frac{z}{{~}^{g}\!\left/ \!{~}_{2}\right.}\right)}^{2}}\end{array}$$

where *z* is the distance between the tape head and the bead, *I* is the applied electric current, *g* is the width of the tape head’s gap, and *A* and *B* are the calibration parameters—that similarly depend on the specific properties of the tape head/bead.

Once this mathematical dependence is established, the calibration parameters can be determined using a molecular quantity with a well-defined force dependence. The most employed method uses the lateral thermal fluctuations of a bead tethered to a long DNA molecule of known length (Strick et al. 1996). According to the equipartition theorem, the pulling force *F* can be calculated from the average fluctuations along a lateral coordinate $\langle \delta {y}^{2}\rangle$, which for a given extension $\langle z\rangle$ of the tether follows:

6 $$\begin{array}{c}F=\frac{kT\langle z\rangle }{\langle \delta {y}^{2}\rangle }\end{array}$$

By measuring the lateral fluctuations $\langle \delta {y}^{2}\rangle$ and DNA extensions $\langle z\rangle$ at different distances between the magnets and magnetic bead, the calibration curve can be determined. Measuring $\langle \delta {y}^{2}\rangle$ presents several practical challenges, both due to external vibrations that can contribute to the molecular fluctuations and the effect of the camera shutter times, which makes it desirable to work on the frequency domain. Additionally, particularly at low forces, the drag coefficient of the bead close to the surface changes non-linearly, which is not reflected in Eq. 6 and is hard to account for, leading to calibration inaccuracies. Discussion on these practical issues and alternative more robust strategies have been excellently discussed in different papers (Pritzl et al. 2024; van Oene et al. 2018; Yu et al. 2014).

Protein (un)folding extension changes have alternatively been employed to calibrate magnetic tweezers instrumentation. When a protein unfolds under force, it responds as an unstructured polypeptide chain whose average end-to-end length rapidly equilibrates to an extension imposed by the pulling force (M. Doi 1995). This relationship can be described by established polymer physics models such as the worm-like chain (WLC) or the freely-jointed chain models (FJC). Conversely, the transition from the unfolded to the folded state results in a corresponding decrease in molecular extension with the same force-scaling behavior.

**Fig. 2:**
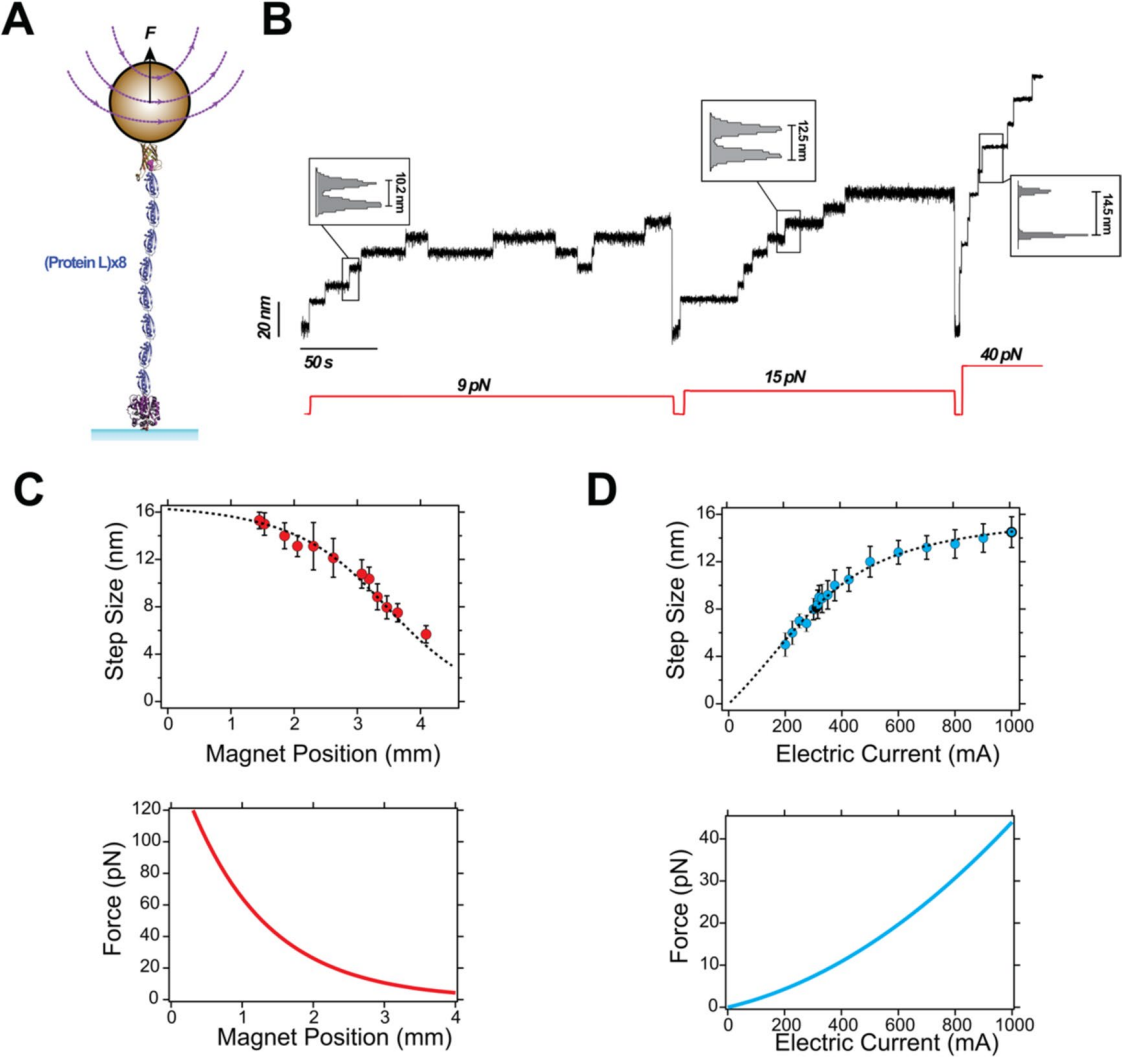
Step-size-based calibration of magnetic tweezers. **A** Schematics of a protein L octamer construct for calibrating magnetic tweezers. **B** Typical recording of a protein L octamer exploring different forces. The step sizes scale with force following the WLC model of polymer dynamics. **C** Calibration for a N52 permanent magnet setup using M270 superparamagnetic beads (*upper)* dependence of the step sizes with the magnet position, fitted to Eq. 4. (*Lower)* Force law. **D** Calibration for a tape-head setup using M270 superparamagnetic beads. (*Upper*) Step size dependence with the electric current, with the tape head placed at a position of 300 µm, fitted to Eq. 5. (*Lower*) Force law

Using the WLC model, the force *F* required to observe an extension change $\Delta z$ in an (un)folding protein can be described as follows (Bustamante et al. 1994):

7 $$\begin{array}{c}F\left(\Delta z\right)=\frac{kT}{{l}_{\text{P}}}\left[\frac{1}{4}{\left(1-\frac{\Delta z}{{\Delta L}_{\text{C}}}\right)}^{-2}+\frac{\Delta z}{{\Delta L}_{\text{C}}}-\frac{1}{4}\right]\end{array}$$

where *l*_P_ is the persistence length of the unfolded polypeptide and ${\Delta L}_{\text{C}}$ is the change in contour length (*i.e*., the unfolded protein’s contour length minus the size of the folded protein). Both *l*_P_ and ${\Delta L}_{\text{C}}$ can be independently determined using other single-molecule techniques, like AFM, which reduces the parameter space and enables accurate calibration. In practice, polyproteins built as tandem repeats of a globular protein are employed for calibration purposes. Protein L has been previously used for this purpose because it unfolds over easily-measurable timescales (a few seconds) across a broad range of forces (~ 4–65 pN) and since its polymer properties have been previously determined (${\Delta L}_{\text{C}}$= 18.6 nm and *l*_P_ = 0.58 nm) (Liu et al., 2009). Figure 2 shows the calibration procedure using protein L step sizes, both for the permanent magnet and tape head instruments. Other classic protein models in force spectroscopy, such as titin’s Ig27 domain, only unfold at very high forces (>100 pN) (Rivas-Pardo et al. 2016), where step size changes are minimal, making them unsuitable for calibration purposes. Similar to the case of fluctuations-based calibration, the sensitivity of the step sizes at high forces decreases, which challenges calibration at high forces (Alonso-Caballero et al. 2021b).

### Design of protein constructs and anchoring chemistry

In the early magnetic tweezers experiments, similar to AFM, unspecific adsorption methods were used to establish single-protein tethers (Liu et al. 2009). However, in AFM, the cantilever tip automatically samples the working surface to locate single molecules. In contrast, magnetic tweezers require the user to manually select and probe individual measuring beads, leading to a markedly low success rate when relying on unspecific anchoring. This limitation has prompted the development of highly specific chemical anchoring strategies to enhance experimental yield.

DNA molecules have been employed conjugated with protein constructs to allow for tethering to the surface and bead, similar to the strategy employed in optical tweezers (Yu et al. 2020). However, long DNA-based tethers introduce large fluctuations that effectively reduce the spatial resolution and therefore are not ideal for protein measurements. In this sense, fusion protein constructs are the preferred strategy in magnetic tweezers, requiring the design of functionalized termini to allow for specific and stable anchors to the bead and surface.

Surface functionalization often starts with the functionalization of the glass coverslip with amine groups using (3-aminopropyl)-trimethiloxysilane, to then crosslink them with glutaraldehyde to amine groups, for example directly using the N-terminus of the protein construct of interest (Dahal et al. 2022). However, this latter strategy lacks specificity as other amine groups exposed in the protein might similarly interact with the surface. More specific approaches involve the use of the HaloTag enzyme at one of the termini of the protein, which allows for covalent anchoring to the surface previously functionalized with the HaloTag chloroalkane ligands (England et al. 2015; Popa et al. 2016)—for instance, the amine O4 ligand enables direct conjugation with glutaraldehyde. A disadvantage of the HaloTag is that it unfolds at high forces (N-terminal HaloTag ~130 pN; C-terminal HaloTag > 400 pN, as measured in force-extension AFM experiments (Popa et al. 2013a), although these forces are lower in magnetic tweezers as slower pulling rates are employed), which introduces artefactual molecular events that can complicate the experiment’s interpretation. Alternative strategies involve “label-free” constructs based on elastin polypeptides that can be used to anchor proteins to the glass surface using terminal cysteine residues (Löf et al. 2019). However, they require in vitro polymerization to concatenate the protein constructs, using sortases, which can be cumbersome to work with in practice. The SpyCatcher/SpyTag split protein technique provides an alternative strategy for in vitro modular concatenation of protein constructs that polymerize spontaneously without the need for enzyme-assisted systems (Dahal et al. 2020; Huang et al. 2022; Zakeri et al. 2012).

Tethering the protein construct to the superparamagnetic bead relies on the choice of the chemistry coating of the bead. The most commonly used beads are streptavidin, amine, or carboxyl coatings, although other chemistries like epoxy or tosyl are also available. Biotinylating one of the protein’s termini that includes an AviTag sequence is one of the most common and simple approaches. However, while the biotin-streptavidin interaction is highly specific and fast (~5 min), it is a non-covalent interaction, which can lead to bead detachment at high forces (> 65 pN), or during very long-term experiments (Löf et al. 2019). Covalent tethering to the superparamagnetic beads overcomes this limitation, for example, by functionalizing amine beads with HaloTag ligands—mirroring the surface chemistry—and using the SpyTag/SpyCatcher system to attach proteins to the bead (Alonso-Caballero et al. 2021a; Tapia-Rojo et al. 2024b).

When designing fusion protein constructs for magnetic tweezers, it is recommended to incorporate some additional protein domains flanking the protein of interest, which separate spatially it from the surface/bead, therefore working as molecular spacers to minimize non-specific interactions. The spacing domains need to ideally remain inert along the experiment and therefore must be proteins with very high mechanical stability, such as stable titin domains (Ig27 or Ig32) or even the inextensible pilin Spy0128 domains (Tapia-Rojo et al. 2024b). Alternatively, elastin-based handles work similarly for this purpose.

## Understanding protein folding dynamics with magnetic tweezers

A major advantage of magnetic tweezers is their intrinsic force-clamp conditions, which allow direct measurement of the protein’s conformational dynamics in equilibrium. This provides a natural interpretation of the measured protein dynamics, without requiring data transformations or corrections by the harmonic force probe, as often necessary in AFM or optical tweezers. Additionally, the precise control of the pulling force and the temporal stability intrinsic to magnetic tweezers allow for a range of force protocols that enable characterizing protein nanomechanics, through both non-equilibrium and long-term equilibrium measurements.

Despite the intrinsic biological interest in understanding the response of proteins to mechanical force, single-molecule force spectroscopy techniques have frequently been used as an experimental method to study protein folding, in an attempt to connect single-molecule behaviors with bulk biochemical experiments. This has driven substantial theoretical work aimed at developing robust frameworks for analyzing and interpreting single-molecule mechanical data, thereby enabling the recovery of the thermodynamic and kinetic quantities governing the underpinning dynamics. However, mechanical force defines a privileged reaction coordinate for the (un)folding transition (the pulling direction), which becomes irrelevant in the absence of force. Furthermore, the mechanical and thermodynamic stabilities of a protein’s folded state are seemingly uncorrelated (Infante et al. 2019), which complicates establishing a direct relationship between folding reactions occurring with and without an applied force.

In this section, we will discuss fundamental physical properties governing protein (un)folding dynamics under force, key theories for interpreting these data, and how magnetic tweezers can be employed to characterize protein dynamics under force.

### Energetic contributions to the (un)folding landscape of a protein under force

**Fig. 3:**
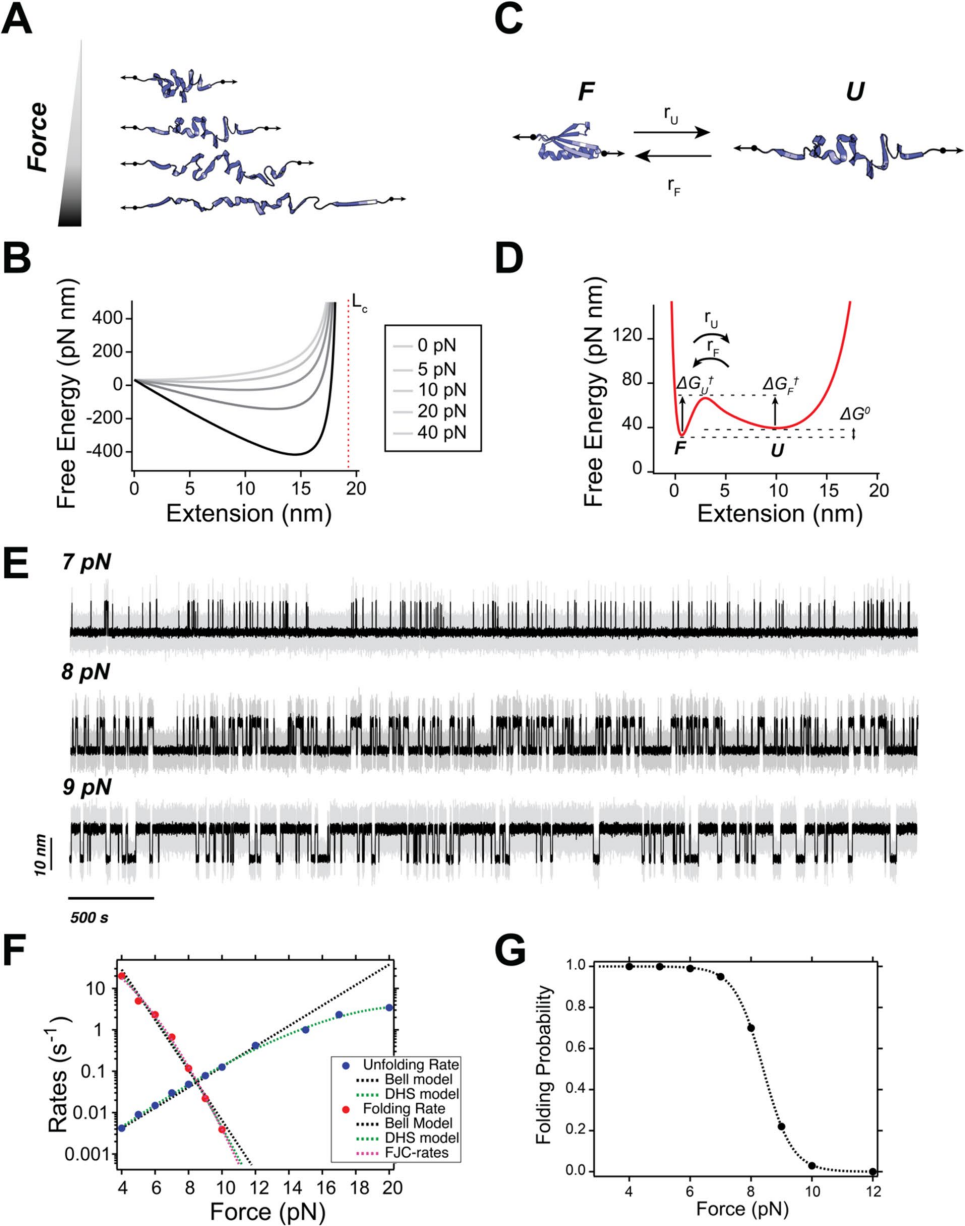
Elements of the energy landscape of a protein folding under force. **A** Schematics of an unfolded protein stretched at different forces. **B** Free energy landscape of an unstructured polypeptide chain at different forces, using ${l}_{\text{P}}$ = 0.58 and ${\Delta L}_{\text{C}}$ = 19 nm. **C** Schematics of a two-state folded-unfolded transition for a protein. **D** Proposed free energy landscape for a folding protein, defining the relevant kinetic and energetic parameters. The parameter set here employed is as follows: *U*_0_ = 50 pNnm, *a* = 1 nm^−1^, *G* = 10 pNnm, *z*_0_ = 2 nm, *s* = 1 nm^−1^, ${l}_{\text{P}}$ = 0.58 and ${\Delta L}_{C}$ = 19 nm. **E** Langevin dynamics simulations over the energy landscape shown in **D**. To mimic experimental recordings, we have added Gaussian noise with a SD = 1.5 nm (similar to the intrinsic noise in magnetic tweezers (Tapia-Rojo et al. 2024a)) and smoothed the recordings with a Savitzky-Golay 4th order algorithm with a box size of 51 points). **F** Folding (red) and unfolding (blue) rates measured from the Langevin dynamics simulations and fitted to different models. Fitting parameters are discussed in the text. **E** Folding probability calculated from the Langevin dynamics simulations

Despite the intrinsic complexity of the protein folding/unfolding reaction, protein (un)folding under force is in most cases well-described by a one-dimensional projection along the pulling coordinate (Neupane et al. 2016b). One key consequence of this force-driven reaction is the significant role of polymer elasticity on the properties of the unfolded protein state (Berkovich et al. 2010b). Upon mechanical unfolding, a protein becomes an unstructured polypeptide chain, stretched by force (Fig. 3A). As a result of this, entropic elasticity governs the energetics of the unfolded state. This concept has crucial implications for protein folding dynamics.

Assuming the WLC polymer model, the free energy profile ${U}_{\text{U}}\left(z\right)$ of an unfolded protein stretched by an applied force *F* can be described by integrating the WLC force-extension relationship* F*_WLC_(z*)*, incorporating the effect of the force as a linear *-Fz* term:

8 $$\begin{array}{c}{U}_{\text{U}}\left(z\right)=\int {F}_{\text{WLC}}\left({z}{\prime}\right)d{z}{\prime}-F\bullet z=\frac{kT}{{l}_{P}}\left[-\frac{1}{4}\frac{\Delta {L}_{\text{C}}^{2}}{z-{\Delta L}_{\text{C}}}+\frac{{z}^{2}}{2{\Delta L}_{\text{C}}}-\frac{1}{4}z\right]-F\bullet z\end{array}$$

Figure 3B shows a plot of Eq. 8 at forces of 0, 5, 10, 20, and 40 pN, using ${\Delta L}_{C}=$ 19 nm and ${l}_{P}$ = 0.58 nm. The force-dependency of the free energy landscape for a polymer under tension has key implications for protein folding dynamics (Fig. 3C). Particularly, the location and energy of the minimum *z*_*U*_ shift with force in a strongly non-linear way (following the WLC), which strongly determines the range of forces over which a protein can refold back into its folded state (Berkovich et al. 2010b, 2010a). Importantly, this description only applies to folding/unfolding reactions occurring under force, where the end-to-end extension is the relevant reaction coordinate—the WLC model expression (Eq. 7) fails as *F→*0 (Marko and Siggia 1995).

By contrast, the native state is defined by the specific, short-range cooperative interactions unique to each protein, which can be modeled using a simple attractive potential, such as a Morse potential (Valle-Orero et al. 2015). Tilting this landscape creates an activation barrier (Berkovich et al. 2018; Valle-Orero et al. 2015), but additional energetic barriers can also be incorporated to account for the short-range entropic interactions associated with the hydrophobic collapse of an amphipathic protein chain in solution (Valle-Orero et al. 2015). A simple Gaussian barrier can be used for this purpose. With this framework, the free energy landscape of a mechanically stretched protein can be described by the following equation:

9 $$\begin{array}{c}U\left(z\right)={U}_{0}\left(1-{e}^{-az}\right)+G{e}^{-\frac{{(z-{z}_{0})}^{2}}{s}}+\frac{kT}{{l}_{\text{P}}}\left[-\frac{1}{4}\frac{{\Delta L}_{\text{C}}^{2}}{z-{\Delta L}_{\text{C}}}+\frac{{z}^{2}}{2{\Delta L}_{\text{C}}}-\frac{1}{4}z\right]-F\bullet z\end{array}$$

where *U*_0_ and *a* characterize the free energy minimum of the folded state (corresponding to the energy and length scales, respectively) and *G*, *z*_0_, and *s* define the free energy barrier (height, position, and width, respectively). Figure 3D shows a plot of this energy landscape, highlighting the relevant energetic and kinetic parameters involving the (un)folding dynamics of a protein under force. At a given force, the unfolding transitions are characterized by the unfolding rates (*r*_U_), which involve overcoming the unfolding free energy barrier Δ*G*^†^_U_, and similarly, the folding rates (*r*_F_) define the inverse kinetic process, involving the folding free energy barrier Δ*G*^†^_F_. The equilibrium free energy Δ*G*°, governing the relative populations of the folded and unfolded state, is simply given by the difference in energy between the position of the folded *F* and unfolded *U* minima.

This free energy description illustrates that, while the parameters defining the unfolding transition are specific to each protein (i.e., the depth and shape of the free energy minimum and height and position of the barrier), the polymer properties of the unfolded protein (given by the persistence and contour lengths) dominate the folding reaction. The persistence length of a polypeptide chain is roughly similar amongst most polypeptide chains (*l*_P_ ≈ 0.5 nm), while the contour length is simply determined by the number of amino acids (which for a typical globular protein range between 50 and 150); by contrast, the folded state (and its stability) can vary enormously for each specific protein. This might explain the very broad spectrum of unfolding behaviors observed in proteins under force—where unfolding forces can be from just a few pN up to hundredths of pN—very different to the narrow range of forces over which proteins typically refold—up to ~12 pN.

### Analyzing and interpreting magnetic tweezers protein folding experiments

Assuming our one-dimensional model for the free energy profile of a protein under force (Fig. 3D), unfolding and refolding transitions correspond to stochastic switching events between the folded and unfolded minima, akin to the classic Kramers’ problem. However, as the landscape is here being dynamically modulated by the mechanical force, these rates will change with force. As force increases, the landscape tilts, favoring the unfolded state and reducing the free energy barrier to unfolding, and vice versa for refolding. According to Kramers’ theory of escape rates over a free energy barrier (in the overdamped and high barrier regime, or Kramers’ regime) (Hänggi et al. 1990; Kramers 1940):

10 $$r\left(F\right)=\frac1{2\mathrm\pi}\frac D{kT}\frac1{\kappa_\cup \kappa_\cap}e^{-\frac{\triangle G^{\dag}\left(F\right)}{kT}}$$

where *D* is the diffusion coefficient, and ${\kappa }_{\cup }$ and ${\kappa }_{\cap }$ are the curvatures of the energy minima and barrier, respectively. Thus, understanding how the escape rates (for either the unfolding or refolding) depend on the pulling force involves determining how the barrier height and curvatures change with force—as the force does not only change the barrier height but might also shift its position.

In a first approximation, we can assume that the barrier decreases linearly with force, such that $\Delta {G}^{\dag}\left(F\right)={\Delta G}_{0}^{\dag}-F\bullet x$, without shifting its position. This approximation describes a scenario with an infinitely high energy barrier, known as the Bell-Evans model (Bell 1978; Evans and Ritchie 1997):

11 $$r\left(F\right)=r_0e^{-\frac{\triangle G_0^\dagger-Fx^\dagger}{kT}}=k_0e^\frac{Fx^\dagger}{kT}$$

where *x*^†^ is the position of the barrier, which modulates the sensitivity of the escape rate to force. Although initially proposed as a phenomenological model, the Bell-Evans model remains the most widely used approach for analyzing folding and unfolding rates in single-molecule experiments, despite its stringent assumptions. The model’s linear approximation generally holds well when only a narrow range of forces is explored, which is often the case in practice. Furthermore, when focusing on unfolding transitions alone, the barrier position *x*^†^_U_ is typically small for most proteins, making the assumptions of the Bell model reasonable.

When assuming more realistic and explicit shapes for the escape barrier, more sophisticated descriptions of the rate-to-force dependence can be derived. Dudko et al. proposed the first expression for the force-activated escape over an analytical landscape, which applied both to a parabolic and a linear-cubic potential. However, as they argued, the linear-cubic potential is the first approximation from a Taylor expansion around the inflection point and captures well the dynamics of most analytical escape barriers. In such a case, the rates can be described as follows (Dudko et al. 2006):

12 $$\begin{array}{c}r\left(F\right)={k}_{0}\left(1-\frac{\nu F{x}^{\dag}}{{\Delta G}_{0}^{\dag}}\right)^{1/\nu-1}{e}^{{~}^{\frac{{\Delta G}_{0}^{\dag}}{kT}\left[1-{\left(1-{\nu F{x}^{\dag}}/{{\Delta G}_{0}^{\dag}}\right)}^{\frac{1}{\nu }}\right]}\ \!{~}}\end{array}$$

where *ν* is a parameter characterizing the shape of the energy profile: with *ν* = 2/3 corresponding to a linear-cubic potential, *ν* = 1/2 a quadratic one, while *ν* = 1 recovers the Bell model. Equation (12) is often known as the DHS model. This expression notably allows for the estimation of the height of the energy barrier in the absence of force, ${\Delta G}_{0}^{\dag}$. Unlike the Bell model’s purely exponential dependence, the DHS model reflects a nonlinear decrease in the height of the energy barrier, along with an associated shift in the barrier position. This results in a curvature in *r*(*F*) that becomes apparent at high forces, where the ${\text{G}}_{0}^{\dag}$→∞ no longer holds.

While the Bell model and the DHS model work well for describing protein unfolding kinetics, as discussed in the previous section, the refolding transition follows a very different landscape that is not well-captured by polynomial descriptions of the energy barrier. As illustrated in Fig. 3D, the position of the unfolded state minimum shifts non-linearly with force, implying that both the barrier height and position for refolding change significantly with force, requiring a different analytical approach to characterize the dependence of the folding rates. Based on first-passage time theory, the escape rate over an arbitrary energy landscape can be expressed as follows (Dudko et al. 2008):

13 $$\begin{array}{c}r\left(F\right)={k}_{0}\text{exp}\frac{1}{kT}{\int }_{0}^{F}\langle {x}^{\dag}\left(f\right)\rangle df\end{array}$$

where $\langle {x}^{\dag}(f)\rangle$ is the difference in the average positions between the transition state and the unfolded state as a function of force. In the case of a refolding protein, we can assume that the entropic elasticity dominates the free energy of the unfolded state, neglecting the contribution from the entropic barrier (Guo et al. 2018). For analytical convenience, we can employ the equivalent FJC model and assume that $\langle {x}^{\dag}(f)\rangle ={x}_{\text{FJC}}(F)$. Under this assumption, the refolding rates are given by the following:

14 $$\begin{array}{c}{r}_{\text{F}}\left(F\right)={k}_{0}\text{exp}\left[-\frac{\Delta {L}_{\text{C}}}{{l}_{\text{K}}}\left(\text{ln}\left|\text{sinh}\left(\frac{F{l}_{\text{K}}}{kT}\right)\right|-\text{ln}\left(\frac{F{l}_{\text{K}}}{kT}\right)\right)\right]\end{array}$$

Interestingly, Eq. 14 has only one free parameter, *k*_0_, since both *ΔL*_C_ and *l*_K_ can be independently measured, and predicts that, essentially, the refolding rates are governed by *ΔL*_C_ and *l*_K_, which act as an effective energy barrier (ignoring the logarithmic terms).

A direct implication of this expression is that shorter proteins should be able to fold against higher force (if *k*_0_ is similar). In other words, the force at which refolding occurs increases as the contour length decreases. This was elegantly demonstrated for the titin Ig27 domain, where the formation of a disulfide bond between Cys32 and Cys75 reduced its contour length from 28.3 to 12.5 nm, which in turn increased the folding forces from 4 to 9 pN (Eckels et al. 2019).

At very low forces the refolding rates dominate, while at high forces, the unfolding rates dominate, leading to a fully folded or unfolded protein equilibrium, respectively. The opposing force dependencies of the folding and unfolding rates imply that there must be a force range over which they are comparable, allowing for measurable coexistent folding and unfolding transitions. For a protein in equilibrium, detailed balance holds, meaning ${r}_{\text{U}}\left(F\right)\bullet {P}_{\text{F}}\left(F\right)={r}_{\text{F}}\left(F\right)\bullet {P}_{\text{U}}\left(F\right)$, where *P*_U_ and *P*_F_ are the populations of the unfolded and folded states. Since *P*_U_ = (1-*P*_F_), the protein’s folding equilibrium can be expressed as follows:

15 $$\begin{array}{c}{P}_{\text{F}}\left(F\right)=\frac{{r}_{\text{F}}\left(F\right)}{{r}_{\text{U}}\left(F\right)+{r}_{\text{F}}\left(F\right)}\end{array}$$

This is known as the folding probability or folded fraction. If force is very low, such that *r*_F_ >> *r*_U_, then *P*_F_ ≈ 1, meaning that the probability of observing unfolding events is vanishingly small. Conversely, at high forces where *r*_F_ << *r*_U_, *P*_F_ ≈ 0, and the probability of observing refolding events is negligible.

To illustrate the validity of our energy landscape model in describing protein folding dynamics under force and the different expressions for analyzing escape rates, we conducted Langevin dynamics simulations at various forces and analyzed the resulting folding and unfolding rates, using the described theories to fit the data (Fig. 3E-G). As discussed before, the Bell model accurately captures the unfolding rates at lower forces, providing a reasonable estimate for the transition state position *x*^†^ = 2.34 nm (the barrier is located 2 nm away from the folded energy well). However, at forces above 15 pN, the non-linear effects of force on the barrier lead to a curvature in the unfolding rates dependence. This effect is better captured by the DHS model, which also yields a reasonable prediction of the barrier’s height and position (${\Delta G}_{0}^{\dag}$ = 53 pNnm and *x*^†^ = 3.12 nm, when the actual barrier height at zero force is ${\Delta G}_{0}^{\dag}$ = 60 pNnm), despite the analytical shape of the potential being quite different from the linear-cubic assumed in the DHS model. For refolding, we observe a similar curvature at low forces, given the shift of the refolding barrier with force. While both the Bell, and particularly the DHS model, provide reasonable approximations of this force dependence, the resulting parameters are difficult to interpret (*x*^†^ =  − 5.8 nm for the Bell model and *x*^†^ =  − 3.35 nm for the DHS model). In contrast, when we fit the data using Eq. 14, it describes the full force-dependency of the rates, also rendering accurate predictions for the values of *ΔL*_C_ = 16.5 nm and *l*_K_ = 0.8 nm as expected from the FJC model (it must be noted that the FJC model predicts shorter contour lengths than the WLC model, compare for instance (Popa et al. 2016) and (Valle-Orero et al. 2015), where the respective contour lengths for protein L are 18.6 nm and 16.3 nm).

### Experimental modes in magnetic tweezers

**Fig. 4:**
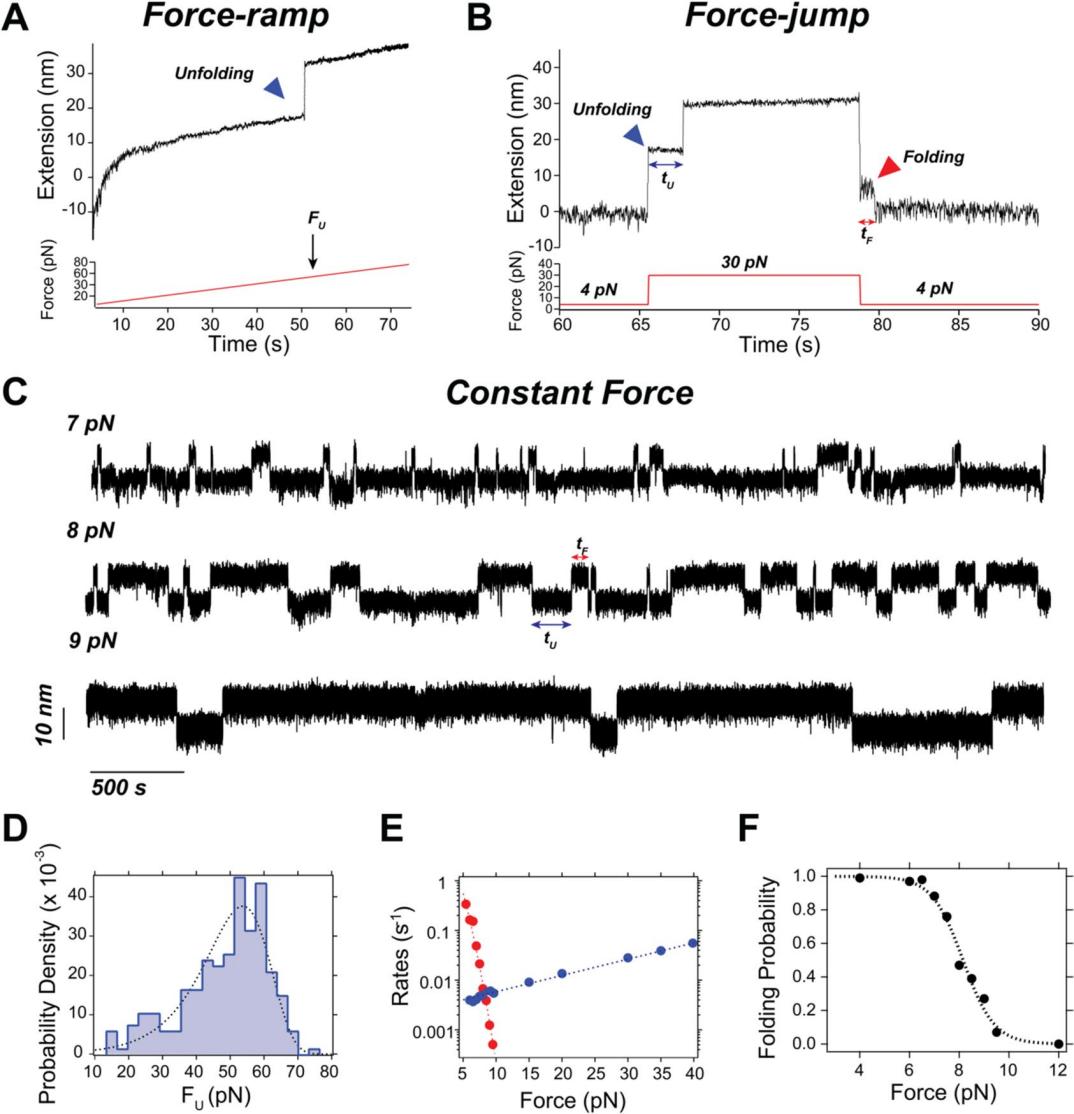
Experimental modes in magnetic tweezers illustrated for a protein L octamer.** A** Typical recording of a force-ramp experiment, where force is linearly increased (here at 1 pN/s), which triggers the non-equilibrium unfolding of the protein, identified as a step-wise increase in the molecular extension. The force at which the unfolding occurs (*F*_U_) is the experimental output. **B** Typical force-jump experiment. Starting at 4 pN with the protein folded, the force is increased to 30 pN, which triggers the unfolding of the protein, identified as a step-wise increase in the protein’s extension; subsequently, the force is reduced back to 4 pN, triggering the refolding of the protein which decreases the protein extension in a step-wise manner. The time to unfold (*t*_U_) and to refold (*t*_F_) are the experimental outputs. **C** Typical trajectories of protein L at different constant forces. Analysis of the dwell times in the folded (*t*_U_) and unfolded states (*t*_F_) allows for calculation of the unfolding and folding rates, respectively. **D** Histogram of unfolding forces as measured from force ramps at a pulling rate of *a* = 1 pN/s, fitted to Eq. 16, yielding ${k}_{U}^{0}=3.3\times {10}^{-3}$ s^−1^ and ${x}_{U}^{\dag}=0.22$ nm. **E** Folding (red) and unfolding (blue) rates as measured from force jump and constant force experiments. The unfolding rates were fitted to a Bell model (Eq. 11), giving $k_U^0=2.8\times10^{-3}$ s^−1^ and ${x}_{U}^{\dag}=0.31$ nm, in fair agreement with the force-ramp data. The folding rates were fitted to Eq. 14, giving ${k}_{0}=9.1$ s.^−1^, *ΔL*_C_ = 16.5 nm, and *l*_K_ = 0.8 nm, in good agreement with the expected values of protein L using the FJC model. **F** Folding probability calculated using Eq. 15. The coexistence force for protein L is *F*_0.5_ = 8.1 pN. Data adapted from Tapia-Rojo et al. (2024a)

Magnetic tweezers offer a variety of force-application modes due to their precise control of the pulling force. Generally, there are three main modes used in magnetic tweezers experiments: (*i*) force ramps, (*ii*) force jumps, (*iii*) constant force. Figure 4 illustrates these commonly used force-protocols pulling, using protein L data as an example.

*Force-ramps* involve the linear increase (or decrease) of the pulling force to trigger the out-of-equilibrium unfolding (or refolding) of the protein, which is observed as a stepwise jump in the molecular extension due to the negligible stiffness of the pulling trap (Fig. 4A). The observed step should correlate with the expected contour length of the protein, providing a molecular fingerprint. This is the most straightforward pulling protocol to interpret, as it enables direct quantification of a protein’s mechanical stability by measuring the unfolding force distribution, being directly comparable to constant-velocity measurements in other techniques, like optical tweezers or AFM—that often involve higher pulling rates. The control parameter here is the pulling rate *a* (in pN/s), where faster pulling rates lead to higher unfolding (or lower refolding) forces, as the unfolding pathway is probed farther from equilibrium. Using the Bell model, the distribution of unfolding forces is given by the following expression (Schlierf et al. 2004):

16 $$\begin{array}{c}{p}_{\text{U}}\left(F\right)=\frac{{k}_{\text{U}}^{0}}{a}{e}^{\frac{F{x}_{\text{U}}^{\dag}}{kT}}{e}^{-\frac{{k}_{\text{U}}^{0}}{a{x}_{U}^{\dag}}\left({e}^{\frac{F{x}_{\text{U}}^{\dag}}{kT}-1}\right)}\end{array}$$

where *k*^0^_U_ and *x*^†^_U _are equivalent to those in Eq. 11. Figure 4D shows the distribution of unfolding forces for a protein L monomer pulled at *a* = 1 pN/s.

Force jumps are another non-equilibrium pulling mode where the force is suddenly stepped between two values, *F*_0_ and *F*_1_. This allows for monitoring non-equilibrium unfolding (if *F*_0_ < *F*_1_ and the protein is folded at *F*_0_) or refolding (if *F*_0_ > *F*_1_ and the protein is unfolded at *F*_0_), which are similarly captured as a stepwise increase (or decrease) in the molecular extension that scales with the protein’s contour length (Fig. 4B). The outcome of these experiments is the unfolding or folding rates, which can be directly determined from the unfolding or folding dwell times (Fig. 4E). Force jumps are employed to characterize the (un)folding kinetics over force ranges where no reversible transitions are observed—either at low forces (where *P*_F_ ≈ 1) or at high forces (where* P*_F_ ≈ 0).

Constant force experiments allow for the measurement of equilibrium dynamics by monitoring reversible folding/unfolding transitions (Fig. 4C). Similar to force jumps, the experimental outcome is the folding and refolding rates, captured as the dwell times in the unfolded or folded state, respectively (Fig. 4E). Additionally, the folding probability can be determined from Eq. 15, or directly measured from the total time the protein spends in the folded state relative to the total measurement time (Fig. 4F). An important quantity describing the folding equilibrium is the coexistence force *F*_0.5_, which can be determined as the midpoint of the sigmoidal dependence of *P*_F_ or as the force at which *r*_F_ and *r*_U_ are equal.

## Applications of magnetic tweezers to study protein dynamics under force

In recent years, magnetic tweezers instrumentation has been increasingly employed in the study of protein dynamics under force. These studies leverage the two primary strengths of magnetic tweezers: their ability to apply low, highly controlled forces, and their capacity to measure molecular dynamics over extended timescales. These capabilities have not only found application in the study of protein folding mechanisms—enabling continuous observation of folding and unfolding transitions over several hours—but have also catalyzed the study of proteins with specialized mechanical functions. The recent bloom in the field of mechanobiology has highlighted the importance of mechanical forces to protein function, particularly for those operating in mechanosensitive cellular structures. Unlike the more classically studied proteins in force spectroscopy (e.g., titin, ubiquitin, protein L), force-sensing proteins have very low mechanical stabilities, responding to forces of just a few pN (Austen et al. 2015; Grashoff et al. 2010), in the range of the forces applied by the actomyosin machinery. Magnetic tweezers have thus found a natural niche in studying these low-stability proteins, where their experimental capabilities become particularly suitable. In addition to protein conformational dynamics, protein binding interactions and chemical modifications can also be regulated by force in many biological instances, and magnetic tweezers can also be implemented in the study of these more complex molecular processes. Below, we review recent studies that showcase the applications of magnetic tweezers in exploring protein dynamics, binding interactions, and force-regulated protein chemistry. It is worth noting that while we provide a targeted overview, the breadth of this field is extensive, with numerous excellent studies that space constraints prevent us from covering in detail.

### Protein folding dynamics

The protein folding problem has long captivated scientists (Dill and MacCallum 2012), and the advent of single-molecule force spectroscopy techniques provided a new perspective to its study. Force-clamp AFM advances led to the first observations of the ability of proteins to reversibly refold after mechanical unfolding (Fernandez and Li 2004); however, the difficulty in precisely manipulating low forces using AFM prevented the direct observation of individual folding events. Optical tweezers have also been widely used to investigate protein folding mechanisms, providing for instance direct reconstructions of free energy landscapes and mapping folding pathways (Neupane et al. 2016b, 2016a). Despite these advances, both techniques are generally limited to a relatively small subset of protein systems, particularly those exhibiting fast reversible folding dynamics that can be captured within seconds-to-minutes-long experimental windows.

In contrast, the recent developments in magnetic tweezers technology have opened up a broader range of possibilities for studying protein folding dynamics, without the temporal constraints in other techniques. While magnetic tweezers provide sufficient resolution to capture fast folding transitions in the millisecond scale (for instance those of the talin R3 domain (Tapia-Rojo et al. 2020)), their strength lies in their capability to resolve folding events in proteins with slow kinetics, such as the titin Ig27 domain (H. Chen et al. 2015a, b; Rivas-Pardo et al. 2016), ddFLN4 (Löf et al. 2019), or protein L (Tapia-Rojo et al. 2024a). This recent evidence has reinforced the idea that protein folding is, in general, a reversible process under mechanical force.

The ability to directly sample refolding dynamics has uncovered unexpected complexities in protein folding mechanisms, challenging classic theories. For instance, one-dimensional models (as discussed in the “Understanding protein folding dynamics with magnetic tweezers” section) predict a monotonic increase of the escape rates with force, as mechanical forces tilt the energy landscape, progressively lowering the energy barrier. However, experiments on octameric constructs of the titin Ig27 domain revealed a non-monotonic dependence, where low forces counterintuitively increase Ig27’s unfolding rates, in a manner resembling a catch-bond-like behavior (Yuan et al. 2017). These findings suggested the presence of a transition state that contracts elastically at low forces, necessitating a second reaction coordinate to account for that unexpected shift in the barrier position. Similar behaviors have been observed for other proteins with comparable structural features (Guo et al. 2020). Furthermore, the ability to rapidly control and change force in magnetic tweezers has enabled direct sampling of folding pathways, revealing mechanisms that depart from classic two-state models. Fast force quenches at low forces have demonstrated that protein L, a well-characterized two-state folder as also described in biochemical bulk experiments, folds through a transient molten-globule-like state (Tapia-Rojo et al. 2019). This immature conformation, populated only for a few milliseconds, parallels previous observations of ubiquitin using AFM (Garcia-Manyes et al. 2009), but occurs at much faster timescales.

The extended timescales now accessible with magnetic tweezers have also allowed the direct observation of rare kinetic events that remain otherwise invisible in shorter experiments. Using the talin R3^IVVI^ protein domain—previously characterized as classic a two-state folder (Tapia-Rojo et al. 2020a)—hour-long magnetic tweezers experiments uncovered an unexpectedly complex conformational space, with up to nine additional conformational states beyond the native folded and unfolded conformations (Tapia-Rojo et al. 2023). Some of these states were linked to proline cis-trans isomerization, a well-known bottleneck in protein folding dynamics (Wedemeyer et al. 2002), while most of them were associated with non-native or misfolded conformations that could temporarily sequester the protein’s dynamics. However, these states could be resolved spontaneously or with force, indicating that they corresponded to distinct conformational states, where the protein was trapped in remote regions of its energy landscapes separated by seemingly high free energy barriers, requiring enhanced sampling to explore them. Overall, these findings—alongside many other studies not mentioned here—highlight a crucial point: one-dimensional descriptions of protein folding dynamics often fail in low-force regimes, where conformational dynamics orthogonal to the pulling coordinate become increasingly relevant.

In addition to the study of the folding mechanism of globular cytoplasmic proteins, magnetic tweezers have also been used to explore the folding pathway of membrane proteins (Choi et al. 2019; Min et al. 2015). Unlike globular proteins that typically fold cooperatively, membrane proteins follow more complex folding pathways, involving the sequential insertion of individual helices into the lipid bilayer, which can be challenging to identify and characterize. Forces around ~25 pN have been shown to be sufficient to completely extract and unfold membrane proteins, which can then be reversibly refolded at low forces of ~4–5 pN, allowing observation of individual insertion events. The complexity of these folding pathways often requires the implementation of advanced step-detection methods, such as Hidden Markov model-like approaches.

Beyond the intrinsic interest of protein folding from a fundamental perspective, the expanding field of mechanobiology highlights the importance of protein dynamics under mechanical force in cellular processes (Beedle and Garcia-Manyes 2023; Yusko and Asbury 2014). Many proteins function within mechanical environments, undergoing force-dependent conformational changes that are essential to their role. Single-molecule force spectroscopy techniques have become crucial for understanding the molecular mechanisms underpinning mechanobiology processes.

Titin, the largest protein in our bodies and the third filament in muscle fibers was amongst the first proteins to be studied using single-molecule AFM and optical tweezers (Kellermayer et al. 1997; Rief et al. 1997). These studies have now been extended using magnetic tweezers instrumentation to explore its mechanical properties, particularly at low forces. Magnetic tweezers have enabled the direct capture of individual refolding events in titin domains (H. Chen et al. 2015a, b; Rivas-Pardo et al. 2016; Yuan et al. 2017), revealing that this seemingly mechanostable protein can refold under force, thus providing a molecular mechanism to deliver mechanical work and contribute to muscle contraction (Rivas-Pardo et al. 2016). Additionally, using a mouse model where the HaloTag was introduced in the titin I-band, native titin molecules were mechanically stretched using magnetic tweezers, allowing the observation of unfolding and refolding transitions on a native protein molecule (Rivas-Pardo et al. 2020). These experiments revealed a mixed population of short- and long-unfolding events, suggesting the presence of disulfide bonds in native titin Ig domains, supporting the potential role of disulfide bonds in regulating titin elasticity, as previous experiments using fusion proteins had indicated (Kosuri et al. 2012).

Proteins involved in mechanotransduction pathways provide fascinating examples of how mechanical forces can regulate protein dynamics to regulate biological processes. Talin, a well-studied mechanosensor in focal adhesions, links integrins to actin filaments and plays a crucial role in regulating cell-matrix adhesions (Gough and Goult 2018). Using magnetic tweezers, its mechanosensitive rod domains were shown to possess hierarchical mechanical stability, with unfolding forces ranging between 5 and 30 pN, enabling it to respond to the varied force signals sensed by cells (Yao et al. 2016). Additionally, its R3 domain, the least stable one, responds to oscillating force signals in an exquisite frequency-dependent manner, adding another layer to the force-sensing ability of this key focal adhesion protein (Tapia-Rojo et al. 2020a). The architecture of cell-cell adhesions, or adherens junctions, mirrors that of focal adhesions, here with cadherins as the extracellular receptors that mediate cell-cell interactions and α-catenin as the adaptor protein crosslinking to actin filaments (Harris and Tepass 2010). The three α-catenin domains, also a vinculin-binding protein like talin, show a hierarchical mechanical stability, unfolding at forces of ~5 pN, ~12 pN, and ~15 pN. The weakest domain shows reversible folding transitions over a physiological range of forces (~4–5 pN) and, importantly, exposes upon unfolding a vinculin binding site, similar to the talin R3 domain (Yao et al. 2014b). In contrast, other force-bearing proteins, like dystrophin—which consists of multiple spectrin-like repeats, a common architecture in other proteins with a mechanical function—display markedly different force responses, with its domains unfolding at high forces and refolding back at much lower forces. This ability to cycle between the unfolded and folded state could enable spectrin-like domains to dissipate mechanical work, operating as mechanical buffers during rapid mechanical deformations (Le et al. 2018).

### Binding to proteins under force

Protein-protein interactions are crucial to biological function, and mechanical forces can provide a molecular mechanism to regulate such molecular interactions. Single-molecule magnetic tweezers have also been employed to characterize binding events in mechanically stretched proteins or to probe the mechanical resistance of biomolecular complexes. The interaction between talin and vinculin is perhaps the paradigm of a force-activated molecular interaction. Early magnetic tweezers studies combined with single-molecule fluorescence demonstrated that vinculin binds to unfolded talin molecules, as the vinculin binding sites in the talin rod domains are cryptic, requiring force to unfold the domains and expose the binding epitopes (del Rio et al. 2009). Subsequent instrumental advancements in magnetic tweezers instrumentation have deepened our understanding of the molecular mechanisms underpinning this interaction (Franz et al. 2023; Tapia-Rojo et al. 2020b; Wang et al. 2021; Yao et al. 2014b, 2014a). Individual vinculin binding events were resolved as a ~3 nm contraction in the unfolded talin protein, indicating that vinculin triggers a coil-to-helix conformational change with important implications for the binding mechanism, establishing an upper limit on the range of forces that allow for vinculin binding (Tapia-Rojo et al. 2020b). While these studies used the talin-binding domain of vinculin (the D1 domain), recent research has explored how these mechanisms apply to full-length vinculin, a much more complex protein that exists in an autoinhibited state (Franz et al. 2023; Wang et al. 2021). While autoinhibited full-length vinculin binds similarly to unfolded talin as the D1 domain alone, the interaction with talin activates vinculin through an intricate allosteric mechanism that weakens the autoinhibition head-to-tail interaction, greatly increasing its affinity (Franz et al. 2023). Although the vinculin-talin interaction enables direct observation of individual binding events due to its marked molecular fingerprint, talin rod domains have many exposed binding sites for other critical focal adhesion proteins, including paxillin, RIAM, or DLC1 (Gough and Goult 2018). Binding events to these sites cannot be directly detected from force spectroscopy recordings. Instead, they are inferred as changes in the protein’s mechanical behavior. For example, the binding of DLC1 to the talin R8 domain was found to greatly stabilize its folded state, rendering a virtually inextensible protein. This suggests a molecular mechanism by which talin’s intrinsic mechanical stability can be modulated through binding interactions, increasing the complexity of talin’s mechanical responses (Dahal et al. 2022).

Beyond the field of mechanobiology, protein binding interactions have also been shown to stabilize protein mechanics, as seen in protein L upon binding of kappa-light chain antibodies (Dahal et al. 2020). Additionally, chaperone activity in proteins under force has also been, particularly in the case of the bacterial trigger factor and DsbA chaperones interacting with protein L (Eckels et al. 2021; Haldar et al. 2017). Both chaperones were shown to increase the ability of protein L to refold under force, likely by interacting with hydrophobic patches exposed upon unfolding, thereby reducing the conformational entropy of the unfolded protein to favor the refolding transition.

In addition to binding interactions in mechanically stretched proteins, protein-protein complexes can be directly subjected to mechanical forces, often to provide structural support in polyprotein linkages. Probing unbinding reactions using single-molecule force spectroscopy is inherently challenging, as molecular unbinding results in an irreversible rupture event, which is undistinguishable from the loss of the molecular tether. To overcome this caveat, a clever molecular strategy, inspired by optical tweezers assays (Kim et al. 2010), is often employed. This approach involves using a fusion protein construct where an unstructured polypeptide linker connects the interacting proteins, enabling binding at low forces, but preventing the loss of the tether upon unbinding, thereby enabling observation of repeated binding and unbinding events. This strategy has been now applied to investigate the interactions of several mechanosensitive protein-protein complexes, including talin-vinculin (Le et al., n.d.), talin-KANK1 (Yu et al. 2019), or α-catenin-vinculin (Le et al. 2024, 2019). These complexes generally follow slip-bond-like force dependences, resisting forces of just a few pN (< 15 pN), yet in the range of the expected forces applied by the actomyosin cellular machinery (Austen et al. 2015). At the other end of the force spectrum, the much stronger interactions between bacterial adhesive proteins and extracellular ligands, which have been extensively studied with AFM (Milles et al. 2018; Milles and Gaub 2020), have also been investigated using magnetic tweezers (Huang et al. 2022). Bacterial adhesins have evolved to form extremely resistant tethers with extracellular ligand proteins, enabling them to withstand the large mechanical stresses encountered at the initial stages of infection. Unlike slip-bond dynamics, where the lifetime of the bond intuitively decreases with force, bacterial adhesins employ more sophisticated mechanisms like catch-bonds, which stabilize under increasing forces. More recently, magnetic tweezers have been used to explore interactions between viral proteins and host proteins, such as the interaction between the COVID-19 spike protein and the ACE2 human receptor (Bauer et al. 2024, 2022). While the role of mechanical forces in the case of viral interactions may seem less obvious, these assays provide a valuable tool for dissecting binding affinities from a single-molecule perspective, which, for instance, has revealed a correlation between higher unbinding forces and the virulency of different COVID-19 variants (Bauer et al. 2024).

### Protein mechanochemistry

The non-obvious role of mechanical forces in regulating chemical reactions in proteins has been a rich area of study using single-molecule techniques, particularly force-clamp AFM. One key example is the role of cysteine reactivity under force as a regulator of titin nanomechanics (Alegre-Cebollada et al. 2014; Beedle et al. 2016; Kosuri et al. 2012). However, the application of magnetic tweezers in protein mechanochemistry remains less explored, with only a few examples largely inspired by previous AFM work. Disulfide bonds are well-described modulators of protein elasticity, reducing the protein’s ability to extend under force and acting as chemical switches to stiffen proteins under force (Kosuri et al. 2012). Magnetic tweezers have extended these studies to evaluate the role of these covalent crosslinking bonds in titin folding. In an engineered mutant Ig27 domain containing two cysteine residues at positions 32 and 75, which enable disulfide bond formation, oxidized proteins were shown to have an increased ability to refold under force, likely due to the effective shortening of the contour length, which reduces the entropic penalty of folding (Eckels et al. 2019). More broadly, oxidative modifications are known to impact protein folding, so most single-molecule experiments must be carried out in a strong antioxidant environment to prevent cumulative changes. This widely accepted notion was directly demonstrated by mechanically stretching individual proteins to expose cryptic residues to a solution in the absence of antioxidants, so reactive oxygen species naturally accumulated over long-term experiments. Protein L, which lacks highly reactive residues like cysteine or methionine, was progressively oxidized when unfolded, losing its ability to refold under force and becoming a mechanically labile polymer. In contrast, protein domains harboring more reactive residues such as the titin I10 and I27 oxidized at much faster rates, although the specific contributions of individually oxidized amino acids could not be pinpointed (Valle-Orero et al. 2017a, 2017b).

Bacterial pilin proteins are particularly attractive for studying protein mechanochemistry due to the range of unique chemical traits they have evolved that confer these proteins with outstanding mechanical stability. Isopeptide bonds are common in the shaft pilin proteins of gram-positive bacteria, preventing their extension (Alegre-Cebollada et al. 2010) or allowing them to operate as potent shock absorbers (Echelman et al. 2016). Similarly, internal thioester bonds found in the tip adhesins of some gram-positive bacteria mediate the attachment of bacteria to lysine residues in host ligand proteins like fibrinogen (Walden et al. 2015). AFM experiments suggested that the reactivity of the thioester bond in the Spy0125 adhesin pilin of *S. pyogenes* was strongly force-dependent (Walden et al. 2015). This was directly measured using magnetic tweezers, establishing that forces above 30 pN hinder the cleavage of this bond, limiting the extensibility of the protein, while lower forces enabled its reversible formation (Alonso-Caballero et al. 2021b).

## Concluding remarks

By leveraging its intrinsic advantages—namely its force-clamp conditions and temporal stability—and overcoming its traditional limitations in spatial and temporal resolution, magnetic tweezers have recently emerged as a crucial technique to study protein dynamics under force. Magnetic tweezers have proven particularly valuable in the study of mechanosensitive proteins, which naturally operate at very low forces, providing a mechanistic understanding of the molecular mechanisms underpinning cellular mechanotransduction. While these studies have been limited to a relatively small subset of protein systems (talin has perhaps become the model protein in magnetic tweezers, akin to the role that titin plays in AFM), the growing recognition of the mechanical roles played by proteins in cellular structures, like focal adhesions, cell-cell contacts, and the nucleus, has now expanded the potential for magnetic tweezers applications. However, a major challenge remains in crossing the scale separation to link these nanomechanical observations to cellular behavior.

Despite significant technological advancements, single-molecule magnetic tweezers applied to proteins have room for instrumental improvement. Time resolution has been a classic limitation, and current magnetic tweezers instrumentation still generally operates at frame rates lower than optical tweezers or AFM. Faster cameras with better signal-to-noise ratios and improved computing strategies (for instance implementing dedicated FPGAs or GPU-based parallel algorithms) could accelerate the image analysis methodology and push the current resolution boundaries. Additionally, while single-molecule fluorescence has been successfully integrated with optical tweezers, the combination of this technology with magnetic tweezers remains less explored, except for a few examples (Ivanov et al. 2018; Kemmerich et al. 2016). Dual force-light measurements could become critical to investigate protein-protein interactions in real-time, a common molecular mechanism in mechanotransduction processes.

## Data Availability

No datasets were generated or analyzed during the current study.
